# Delayed Splenic Rupture Secondary to Chronic Staple Line Leak Following Laparoscopic Sleeve Gastrectomy: A Case Report

**DOI:** 10.7759/cureus.78697

**Published:** 2025-02-07

**Authors:** Justin M Hsieh, Gabriel Land, Nariyoshi Miyata, Tasmea Sefa, Francis Asomah

**Affiliations:** 1 Department of Surgery, Mount Isa Hospital, Mount Isa, AUS

**Keywords:** bariatric surgery complications, delayed postoperative complication, endoluminal vacuum therapy, laparoscopic sleeve gastrectomy, splenic rupture, staple line leak

## Abstract

Laparoscopic sleeve gastrectomy (LSG) is one of the most frequently performed bariatric procedures worldwide due to its efficacy in achieving significant and sustained weight loss. While generally safe, rare but life-threatening complications can occur. This case report describes a 54-year-old female who presented with acute left-sided abdominal pain and hemodynamic instability six months after an otherwise uneventful LSG. She was found to have a delayed spontaneous splenic rupture secondary to a chronic gastric staple line leak. Following urgent surgical intervention with splenectomy, the patient’s condition initially improved but persistent abdominal collections and ongoing pain prompted further investigation, ultimately revealing a small staple line defect. Endoluminal vacuum therapy (EVT) and prolonged intravenous antibiotics were required. This case emphasizes the importance of maintaining a high index of suspicion for delayed postoperative complications such as chronic staple line leaks and their potential to precipitate rare events like spontaneous splenic rupture. Early recognition, prompt surgical intervention, and appropriate multidisciplinary management are critical in preventing catastrophic outcomes.

## Introduction

Laparoscopic sleeve gastrectomy (LSG) has become the most commonly performed bariatric surgical procedure worldwide, accounting for more than 50% of all primary bariatric surgeries in many series [1,2]. Its popularity is attributed to favorable outcomes in terms of weight loss and comorbidity resolution, as well as a relatively favorable safety profile compared to other bariatric procedures [1-3]. Nevertheless, LSG is not without complications. Common adverse events include staple line leaks, bleeding, strictures, and gastroesophageal reflux disease (GERD) [1,3,4]. A chronic gastric staple line leak is defined as a persistent defect along the surgical staple line that fails to seal properly in the postoperative period, typically beyond two weeks after surgery [1]. These leaks arise when factors such as local ischemia, excessive tension on the staple line, or technical issues during the creation of the staple line impair proper healing. The incidence of gastric staple line leaks is generally reported to be between 0.7% and 3%, while splenic injuries occur in approximately 0.2% of cases [4-7].

Most splenic injuries associated with LSG are usually identified intraoperatively or in the early postoperative period, typically within the first 48 to 72 hours after surgery [5]. It is often attributed to direct trauma during mobilization of the fundus and short gastric vessels [6,7]. Delayed splenic rupture unrelated to immediate surgical manipulation is exceedingly rare and has only been infrequently reported [8]. This case report presents a unique scenario in which a chronic gastric staple line leak resulted in an insidious inflammatory process that ultimately led to delayed splenic rupture six months following an LSG. It highlights the need for ongoing vigilance for late complications and highlights the importance of early diagnostic imaging and multidisciplinary management in patients with unexplained abdominal pain following bariatric surgery.

## Case presentation

A 54-year-old female with a history of type 2 diabetes mellitus, hypertension, ischemic heart disease, and obesity underwent an LSG at a regional hospital. The initial procedure was uneventful, with no immediate postoperative complications. Six months later, she presented to the emergency department (ED) of a remote hospital with severe, sudden-onset left-sided abdominal pain radiating across her abdomen, associated with nausea and hypotension. She reported intermittent, low-grade abdominal pain since her LSG but had not sought medical attention for these issues until this acute presentation.

On examination, the patient appeared pale, diaphoretic, and hemodynamically unstable with a blood pressure of 90/60 mmHg and tachycardia. She had generalized abdominal tenderness without focal peritonism. Initial laboratory investigations on presentation to ED showed low hemoglobin and markedly elevated inflammatory markers (Table 1).

**Table 1:** Laboratory investigations at index presentation to ED ED, emergency department

Observation	Value	Units	Reference Range
Hemaglobin	97	g/L	115-160
White Cell Count	28.9	x10^9^/L	4.0-11.0
C-Reactive Protein	416	mg/L	<5.0

**Figure 1:**
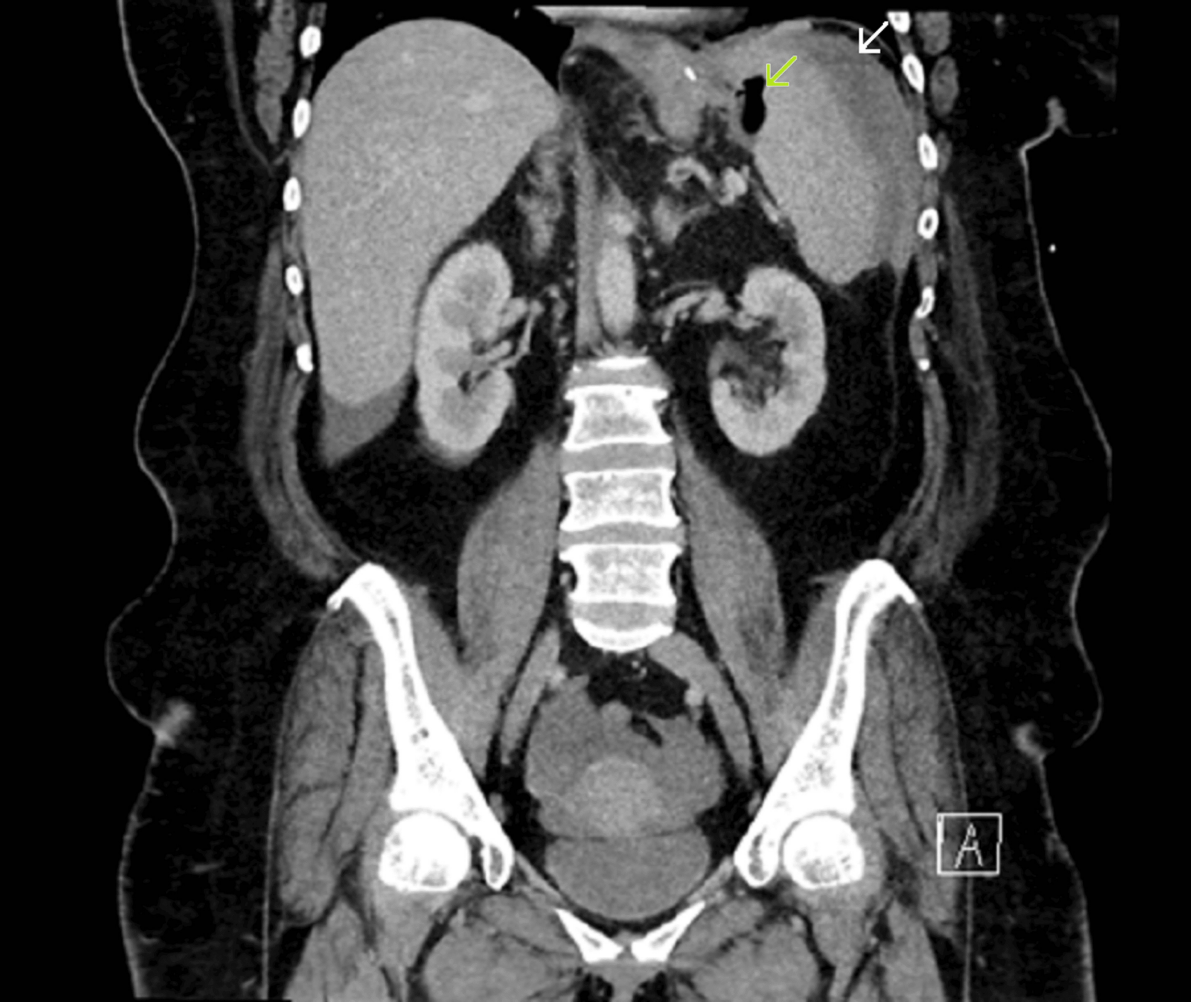
Index CT scan on presentation to ED Green arrow indicating gas surrounding splenic hilum. White arrow indicating intra-abdominal free fluid. ED, emergency department; CT, computed tomography

A contrast-enhanced computed tomography (CT) scan revealed moderate intra-abdominal free fluid and a suspicious gas tract extending from the gastric staple line toward the splenic hilum (Figure 1).

An urgent diagnostic laparoscopy was performed with a presumptive diagnosis of a staple line leak. Intraoperatively, a moderate hemoperitoneum was encountered, but there was no obvious gastric fluid or frank contamination. No immediate source of bleeding was identified, and the patient was admitted to the intensive care unit for close monitoring.

**Figure 2:**
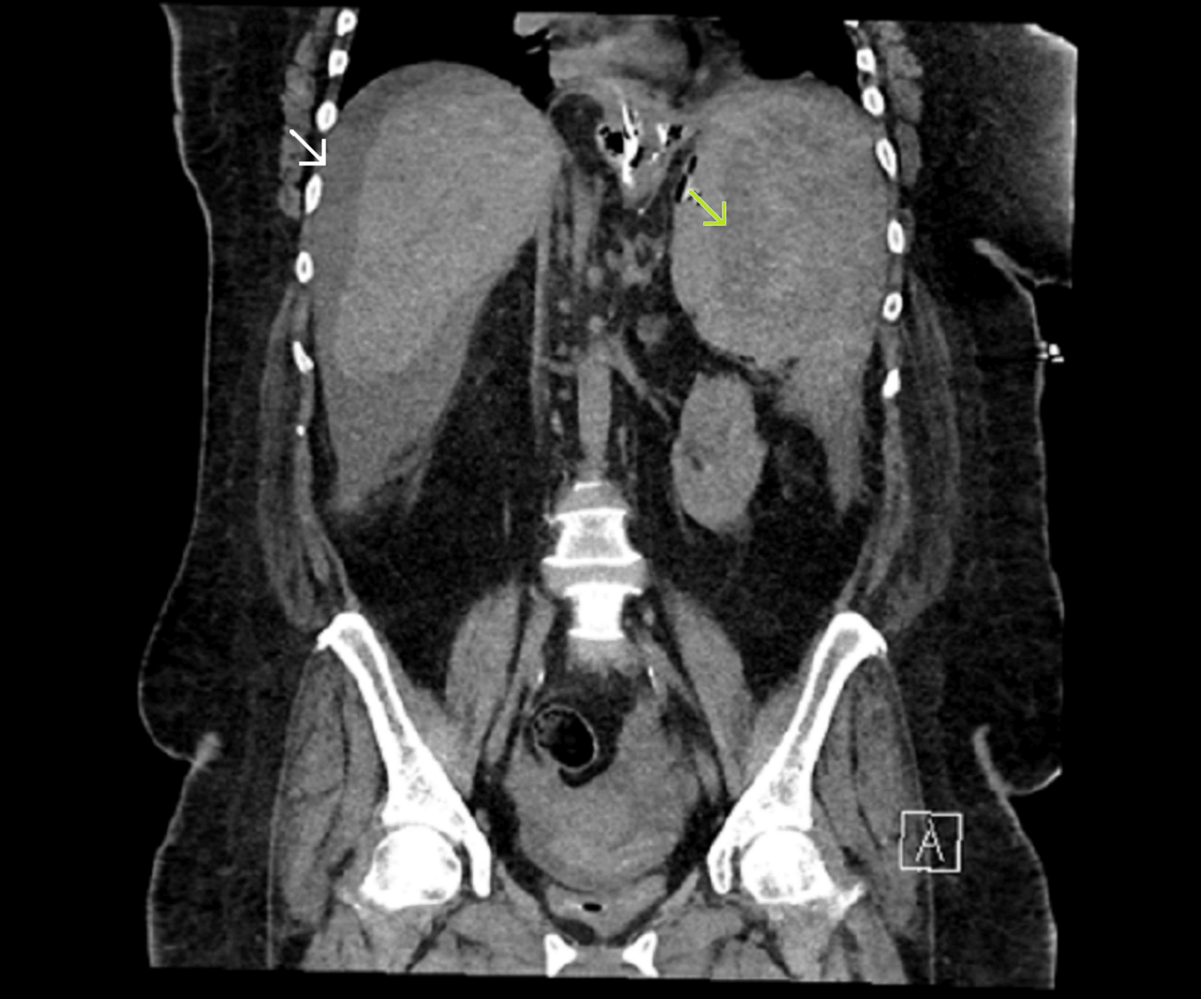
Repeat CT scan, day one post-diagnostic laparoscopy Green arrow indicating ruptured splenic capsule. White arrow indicating increasing intra-abdominal free fluid. CT, computed tomography

Over the next 12 hours, the patient remained persistently tachycardic, and her hemoglobin levels failed to increase despite multiple blood transfusions. A repeat CT scan showed increasing intra-abdominal fluid, especially along the left paracolic gutter (Figure 2). An urgent exploratory laparotomy revealed a ruptured spleen with ongoing hemorrhage. A splenectomy was performed to achieve hemostasis. The patient’s condition initially stabilized, and her clinical parameters improved.

**Figure 3:**
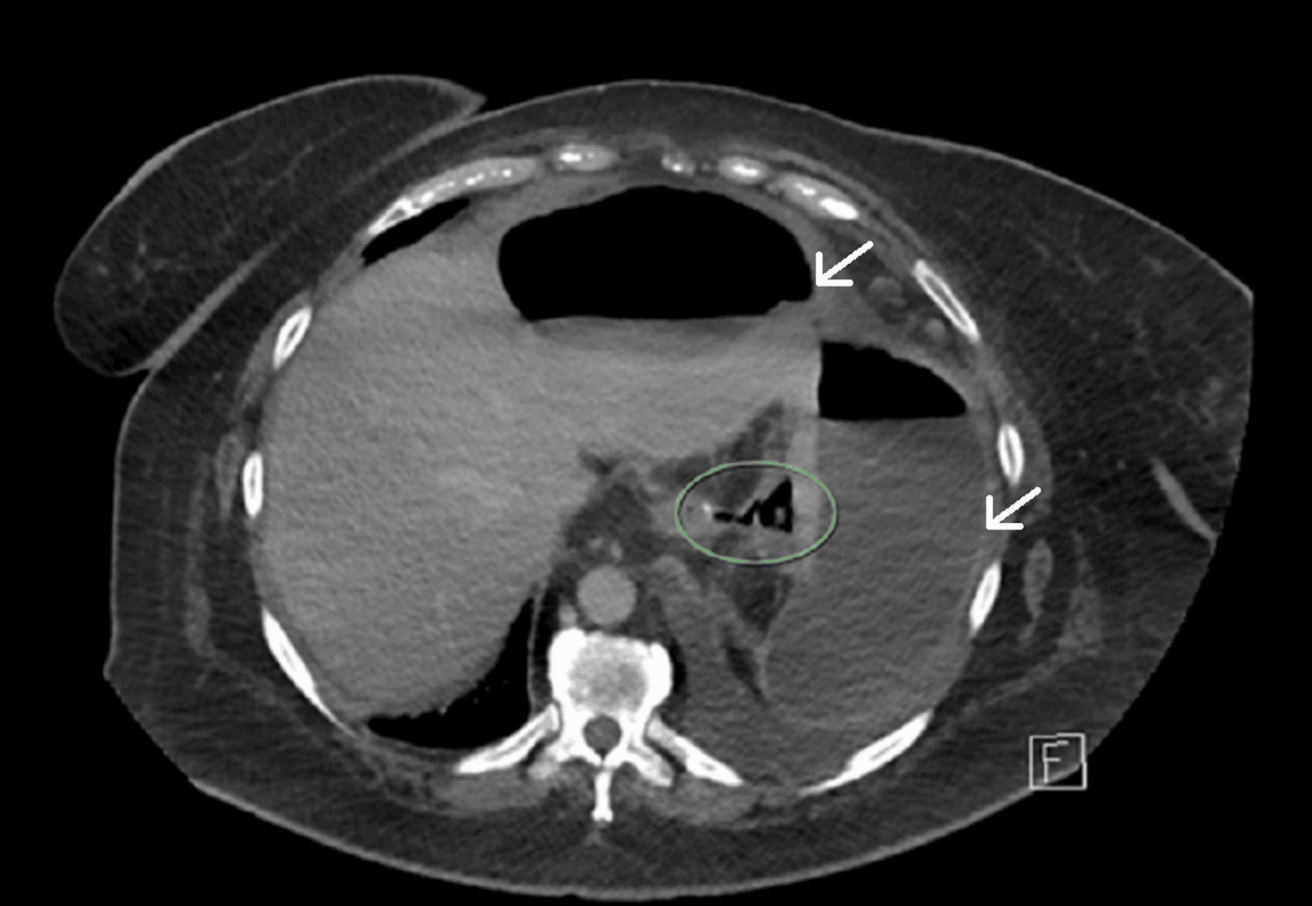
CT scan of intra-abdominal collection, day six post-splenectomy Green circle highlighting the source of gastric sleeve leak at the superior aspect of the staples line. White arrows indicate dumbbell-shaped intra-abdominal gas and fluid collection. CT, computed tomography

However, by the sixth postoperative day, she developed worsening abdominal pain and a persistent elevation in CRP, and imaging revealed a recurrent fluid collection (Figure 3). Broad-spectrum intravenous antibiotics were administered, and she was transferred to a tertiary center under the upper gastrointestinal surgical subspecialty team for further management. 

**Figure 4:**
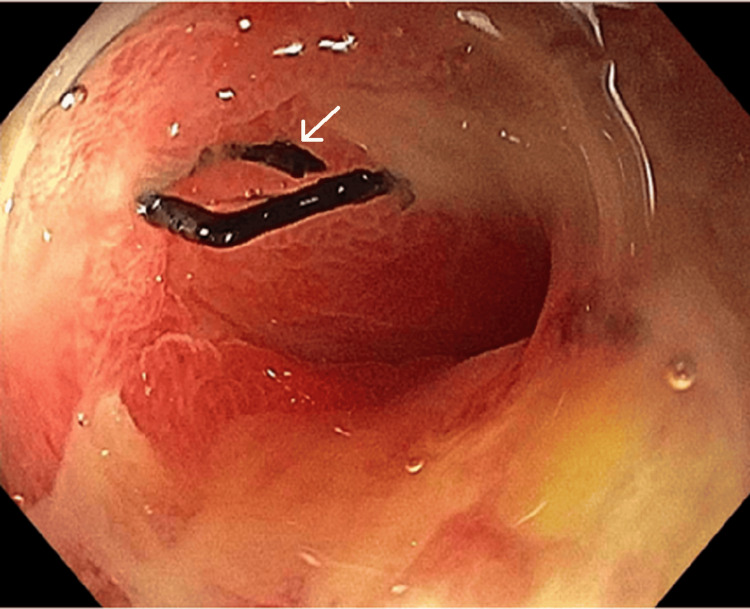
Endoscopic view of gastric sleeve leak with suture material on view White arrow indicating black suture material on view through a pinpoint defect in the superior aspect of the staple line.

At the tertiary center, a gastroenterologist performed the endoscopy under sedation. The endoscopic evaluation identified a small 5 mm staple line leak with black suture material on a view (Figure 4). A polyurethane endoluminal vacuum therapy (EVT) sponge was endoscopically positioned over the leak site and was connected to a vacuum pump set to deliver continuous negative pressure at -150 mmHg. In this case, EVT was maintained for a duration of 18 consecutive days. Subsequent repeat imaging and endoscopic evaluation confirmed the eventual resolution of the leak.

The patient’s condition gradually improved following a combination of EVT and antimicrobial therapy. She was eventually discharged on a tailored rehabilitation program. This coordinated approach facilitated effective infection source control and contributed significantly to the patient's recovery.

## Discussion

Splenic injury is a well-documented but relatively uncommon complication of LSG, predominantly occurring during the operative phase due to inadvertent trauma to the spleen or its vasculature [4,5,7]. Postoperative splenic hemorrhage or rupture occurring weeks to months after surgery is exceedingly rare. While there are reports of spontaneous splenic rupture in association with various underlying conditions, including portal vein thrombosis or subcapsular hematomas, very few cases have been described in the context of LSG [8,9].

Chronic staple line leaks pose significant challenges, occurring in approximately 1-3% of LSG cases [4]. They often present with subtle, nonspecific symptoms such as mild abdominal pain, malaise, or elevated inflammatory markers. Left untreated, these leaks can lead to abscess formation, fistulae, or chronic inflammatory changes in surrounding tissues. In this case, the presence of a persistent, low-grade leak likely caused ongoing inflammation in the left upper quadrant, weakening local tissues and contributing to the eventual rupture of the spleen.

The delayed splenic rupture, in this case, is suspected to have resulted from the same complications typically seen with untreated chronic gastric staple line leaks. Persistent chronic inflammation, progressive tissue erosion, and local abscess formation adjacent to the splenic hilum likely compromised the integrity of the splenic capsule over time, ultimately predisposing it to rupture. Although the exact pathophysiological process remains speculative, the temporal relationship between the onset of the chronic leak and the subsequent splenic rupture strongly suggests that these inflammatory and erosive processes were the principal contributing factors in this patient’s delayed splenic rupture.

Given the rarity and complexity of this presentation, a high index of suspicion is essential. Clinicians managing patients who present with unexplained abdominal pain, hemodynamic instability, and a history of bariatric surgery should immediately consider imaging studies to rule out unusual complications. Multi-phase CT imaging can provide critical diagnostic clues by identifying fluid collections, gas tracts, or subtle organ injury [3-5]. Early surgical or endoscopic intervention is often warranted, as delayed management may lead to life-threatening outcomes.

Furthermore, prevention strategies should include meticulous surgical technique, careful dissection around the short gastric vessels, and the routine use of postoperative imaging for leak detection. Although discussion of these protocols is beyond the scope of this case report, adherence to standardized surgical protocols and diligent postoperative follow-up may help in the early detection and prevention of such complications. Surgeons should still remain vigilant even in patients presenting many months after LSG with abdominal pain and signs of sepsis or hemorrhage.

## Conclusions

This case illustrates a rare but life-threatening complication of LSG: a delayed spontaneous splenic rupture secondary to a chronic staple line leak. As LSG continues to be performed in increasing numbers globally, it is critical for clinicians to maintain a high index of suspicion for atypical and delayed complications. Prompt recognition through prompt imaging and early endoscopic evaluation, including the potential use of EVT, may help mitigate the progression of complications and reduce the need for subsequent surgical takebacks to the theatre. Timely diagnosis and intervention, whether surgical or endoscopic, are essential in minimizing overall morbidity and mortality. Further research is needed to understand the pathophysiological mechanisms underlying such rare complications and to develop strategies for early detection, improved prevention, and targeted management.
